# Anesthetic manipulation in extreme airway stenosis: a case report

**DOI:** 10.1186/1752-1947-8-292

**Published:** 2014-09-04

**Authors:** Zhi-Bin Zhou, Xiao-Yu Yang, Xue Zhou, Shi-Hong Wen, Ying Xiao, Xia Feng

**Affiliations:** 1 Department of Anesthesiology, First Affiliated Hospital of Sun Yat-Sen University, No. 58 Zhongshan 2nd Road, Guangzhou 510080, PR China

## Abstract

**Introduction:**

Anesthetic management with airway stenosis is challenging. Techniques for maintaining spontaneous respiration are required under sedative and analgesic conditions.

**Case presentation:**

A 35-year-old Chinese woman presented to our hospital with difficulty breathing. Computerized tomography showed a tumor in the frontal area of her neck, which was causing extreme narrowing of her trachea. She was immediately scheduled for emergency surgery to remove the tumor. Fiberscopic intubation was carefully performed with dexmedetomidine sedation and remifentanil analgesia. Spontaneous respiration was successfully maintained.

**Conclusion:**

In cases of extreme airway stenosis, intubation can be safely achieved with dexmedetomidine sedation and remifentanil analgesia.

## Introduction

Anesthetic management in cases of airway stenosis is challenging, and it is crucial to fully evaluate the airway preoperatively. Plain computerized tomography (CT), chest X-ray, laryngoscopy and further pulmonary function tests are often employed to assess airway condition. Techniques for maintaining spontaneous respiration under sedative and analgesic conditions are required. Most sedatives and analgesics suppress respiration and put such patients in peril.

We managed a patient whose airway was severely narrowed due to the metastasis of a thyroid tumor. Sedative intubation was attempted and successfully accomplished with a fiberscope.

## Case presentation

**Figure 1:**
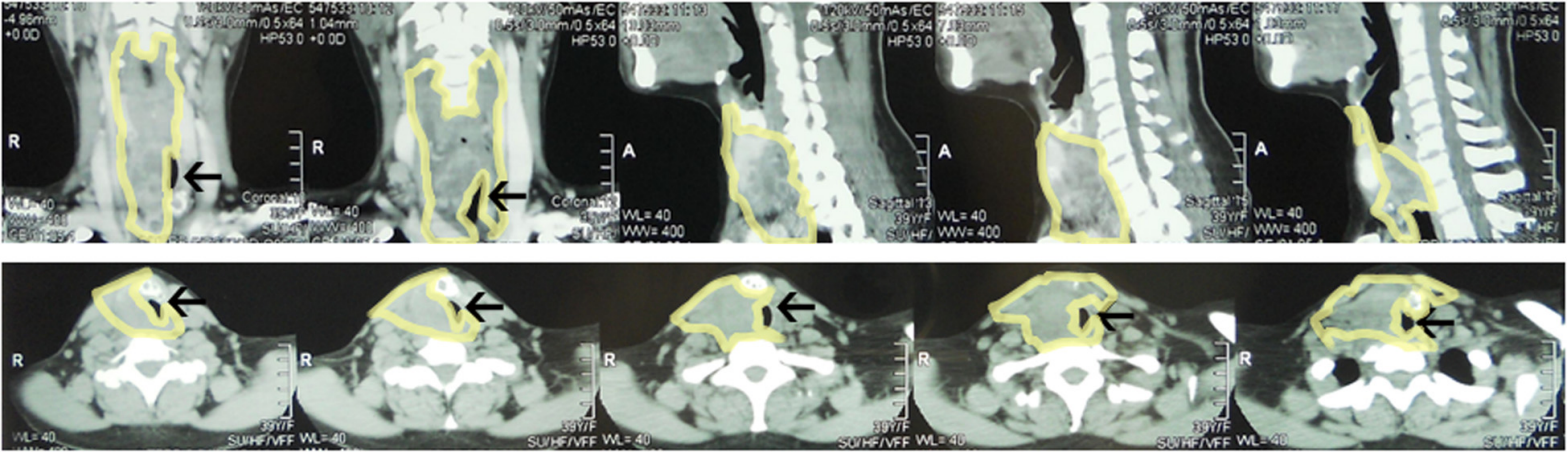
**Computerized tomography scan showed severe stenosis of the airway.** The area within the yellowish border denotes the distribution of the tumor. The remaining airway is indicated by the black arrow.

**Figure 2:**
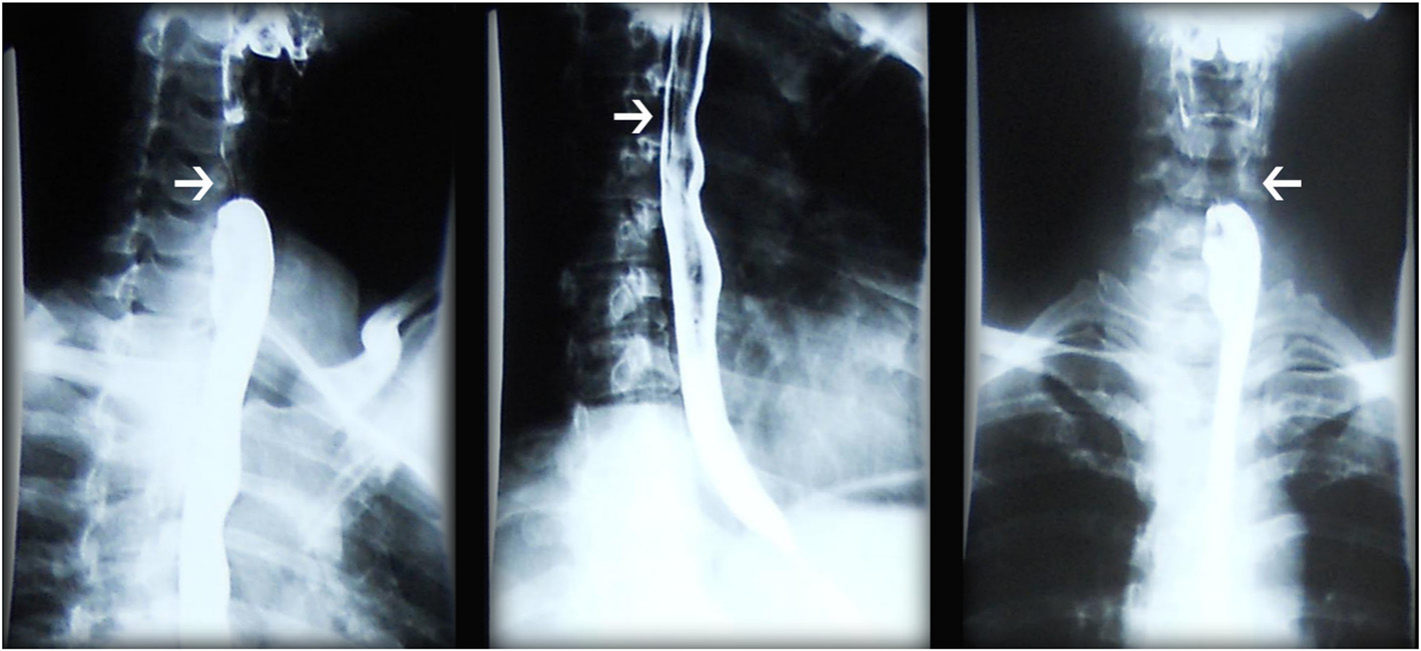
**X-ray showed compression of the esophagus.** The white arrow indicates the compressed part of the esophagus.

A 35-year-old Chinese woman (weight 56kg, height 158cm) discovered a lump on the anterior surface of her neck one month prior to her presentation. Thereafter, she progressively developed difficulty swallowing and breathing. A CT scan and X-ray indicated metastasis of thyroid cancer in and around her trachea, encompassing most of the anterior cervical region and compressed her trachea and esophagus to the left, leaving only a very narrow airway (Figures 1 and 2).

**Figure 3:**
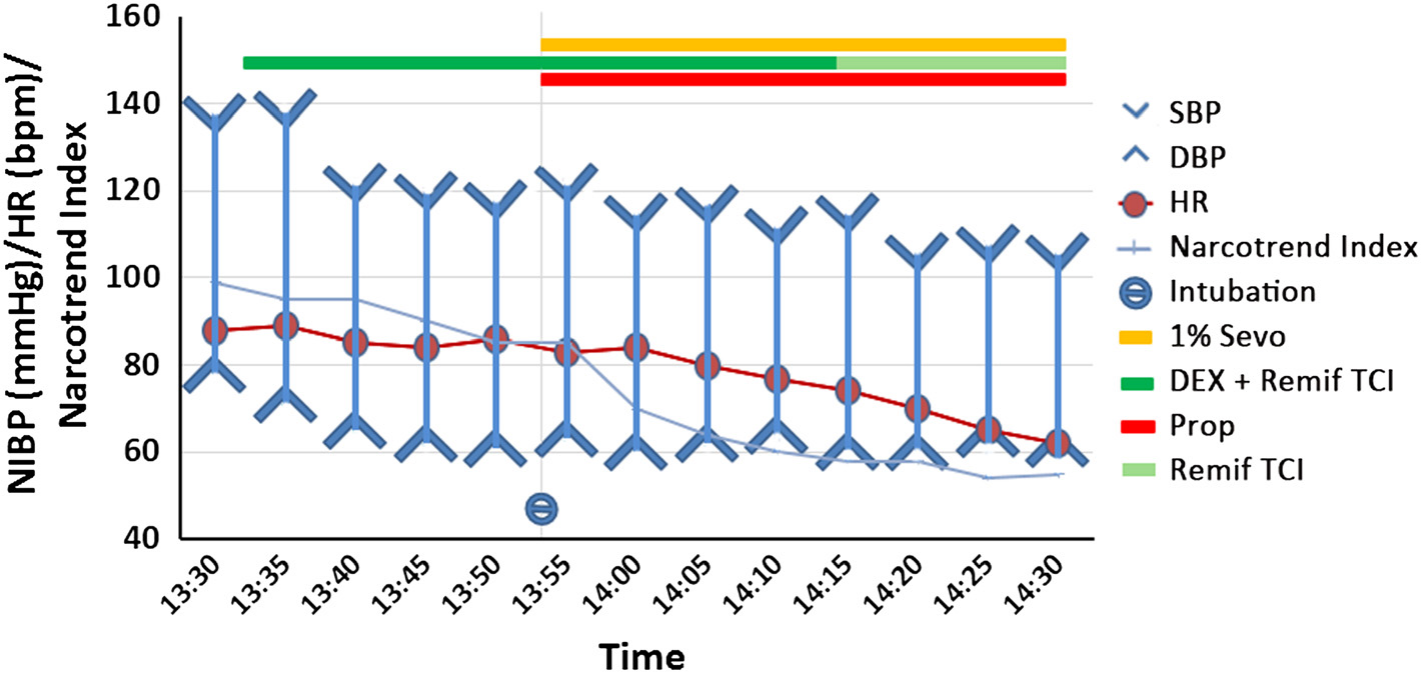
**Patient’s vital signs and sedation level with Narcotrend® electroencephalography analysis.** The Narcotrend Index is an electroencephalography (EEG)-derived index ranging from 1 to 100, indicating sedation level from no EEG activity to actively awake, as follows: 70 to 90, sleeping or mildly sedated; 40 to 70, anesthetized. DBP, diastolic blood pressure; HR, heart rate; NIBP, non-invasive blood pressure; Prop, propofol; Remif, remifentanil; SBP, systolic blood pressure; Sevo, sevoflurane; TCI, target controlled infusion.

Surgery to remove the tumor and reconstruct the airway was immediately required. However, the major difficulty facing our surgical team was the method for safely establishing an airway before anesthesia. Because the tumor was primarily located at the C7 to T2 level, a tracheotomy could not be performed. Our patient had a Mallampati class zero airway, a thyromental distance of 5cm wide and a mouth opening of 4cm wide. We expected little difficulty exposing her vocal cord. According to the CT measurement of the narrowest section of airway opening, an endotracheal tube with inner diameter 5.0mm was chosen to ensure passage of the tube.Dexmedetomidine and target controlled infusion (TCI) remifentanil were infused at rates of 0.5μg/kg/h and 3ng/ml, respectively. Topical anesthesia was applied with 2% lidocaine over her nasopharynx, oral pharynx and laryngopharynx. After 25 minutes, she was properly sedated to endure airway stimulation. Nasal fiberscopic endotracheal intubation was attempted and achieved in spite of a contorted, difficult airway. The tumor under the vocal cord was rubbery with a mobile nature. Spontaneous respiration was maintained successfully. No hypoxia or adverse airway reflex was noted during the procedure. Vital signs were stable (Figure 3). After confirmation of successful airway establishment, sevoflurane inhalation and intravenous propofol were quickly administered to deepen her sedation level. A muscle relaxant was provided to facilitate mechanical ventilation. The subsequent tumor removal surgery was performed and patient woke up breathing with ease.

## Discussion

Preoperative evaluation of the airway is essential in patients with an abnormal airway. Multi-slice CT, which can be reconstituted with three-dimensional (3D) images, can provide a virtual bronchoscopic view [1]. However, because our patient presented as an emergency case, a 3D-image reconstruction was not performed. All efforts were made to release the airway obstruction as soon as possible.

When confronted with a difficult airway before general anesthesia, the most routine maneuver is awake intubation. In awake intubation, no neuromuscular blocking drug is administered to minimize the risk of a failed airway. However, the entire process is too distressing, because only topical anesthesia of the nasal and laryngeal area is applied. With proper sedation, the procedure is more tolerable. The commonly used sedatives include propofol, sevoflurane and dexmedetomidine. Sevoflurane is equivalent to propofol for the performance of fiberoptic intubation under spontaneous respiration in terms of the success rate, patient recall and satisfaction [2]. However both sevoflurane and propofol suppress respiration to a certain extent; if not skillfully titrated individually, patients will either be agitated or the airway easily compromised [3]. In a study that compared propofol with dexmedetomidine for sedation during fiberoptic nasal intubation, patients in the dexmedetomidine group experienced a lower heart-rate response to intubation, showed better tolerance, and had a more stable hemodynamic status [4].

In addition to sedation, remifentanil can be used to relieve pain and blunt nerve reflexes during intubation. Because of its short duration of action, the risk of respiratory depression is minimized. In our previous study [5], TCI remifentanil and TCI propofol were compared in awake fiberoptic intubation. TCI remifentanil at 4ng/mL provided better conditions for opening the vocal cord and shortened the duration of the intubation procedure. As a result, in this case, we used dexmedetomidine and remifentanil together to provide better airway exposure and improve our patient’s hemodynamic safety and comfort.

The instruments used in an anticipated difficult airway include a fiberscope, video laryngoscope and light wand. In our case, although a Macintosh laryngoscope could easily have exposed her vocal cord, we expected to find it difficult to enter her trachea. Thus only a fiberscope was feasible to obtain a view of the airway in her trachea.

Another approach for a difficult airway is invasive access to enable ventilation before anesthesia. The American Society of Anesthesiologists difficult airway algorithm defines invasive access as a surgical or percutaneous airway, jet ventilation and retrograde intubation [6]. However, for our patient, because the tumor was located under her vocal cords and covered the anterior cervical region, neither cricothyrotomy nor tracheotomy could be performed.

As a last resort, extracorporeal membrane oxygenation (ECMO) has also been used in situations where ventilation cannot be performed. ECMO can provide support for severe respiratory failure [7,8]. The advantage of ECMO is that gas exchange can be entirely supported during surgical procedures on the airway while also providing an apneic unobstructed surgical field [9]. ECMO can successfully circumvent the problem of a difficult airway and avoid the risks of sudden airway compromise when an immediate tracheotomy is not possible [10].

A highly specialized team of ear, nose and throat specialists, thoracic surgeons, anesthesiologists, and operative supporting staff is required in cases of difficult airways or surgeries on the trachea and carina. Malignant lesions involving the carina and tumors arising from the lung with radial immersion of the main bronchus and carina remain a challenge in airway management, both in terms of surgical and anesthetic consideration. Every so often, pneumonectomy, carinal resection and complicated reconstruction are required to establish airway continuity. Careful assessment and operative planning by both the surgeons and anesthesia team are essential for optimal results [11].

## Conclusion

In cases of extreme airway stenosis, intubation can be safely achieved with dexmedetomidine sedation and remifentanil analgesia.

### Consent

Written informed consent was obtained from the patient for publication of this case report and accompanying images. A copy of the written consent is available for review by the Editor-in-Chief of this journal.

## Competing interests

The authors declare that they have no competing interests.

## Authors’ contributions

ZBZ did literature research and drafted the manuscript. XYY participated in coordination and helped prepare the manuscript. XZ collected the patient’s data and did the graphical design. SHW obtained the patient’s CT scan and X-ray photo and helped with graphical design. YX assisted with topical anesthesia and intravenous drug administration and revised the manuscript. XF performed the intubation and did the final revision of the manuscript. All authors read and approved the final manuscript.
